# Diversity in scientific publishing is a strategic contribution to plural science

**DOI:** 10.1590/0102-311XEN200825

**Published:** 2025-12-01

**Authors:** Luciana Dias de Lima, Suely Deslandes

**Affiliations:** 1 Escola Nacional de Saúde Pública Sergio Arouca, Fundação Oswaldo Cruz, Rio de Janeiro, Brasil.; 2 Instituto Nacional de Saúde da Mulher, da Criança e do Adolescente Fernandes Figueira, Fundação Oswaldo Cruz, Rio de Janeiro, Brasil.

“*What are we talking about?*” - this was the question with which CSP opened the panel *Diversity and Inclusion in Scientific Publishing* during the seminar organized by the Abrasco Public Health Editors Forum held in São Paulo, Brazil, on June 12 and 13, 2025.

This seemingly simple question led us to an urgent, complex discussion: the persistence of structural inequalities that permeate scientific production and communication. The issue is not peripheral. It addresses thinking about who has a voice in science, who decides what is published, and what knowledge is legitimized in editorial spaces in diverse, unequal societies.

The importance of this discussion resides in understanding that scientific publishing is not merely a technical forum for assessing and disseminating knowledge. Nor is it a neutral space. It is also a field of symbolic, social, and political disputes, where inequalities of gender, race, class, region, and language are legitimized ^1^. Recognizing these asymmetries is an essential step towards transforming them in order to enable more pluralistic and inclusive editorial practices.

Five major axes structure this discussion and pose challenges for scientific journals. The first regards the structural dimensions of inequality manifested in the systematic exclusion of particular social groups and the linguistic barrier imposed by the hegemony of English. This limits the circulation of production from the Global South and undervalues knowledge in other languages ^2^.

The second axis refers to gender, race, and regional inequalities, expressed in the invisibility of black, Indigenous, and trans researchers ^3^ and in the geographic concentration of scientific production. In Brazil, this concentration reflects the distribution of the main research centers and graduate programs.

The third axis addresses the role of editors and reviewers involved in the assessment process and the decision of what is publishable. It is necessary to recognize that editorial boards still have a low degree of diversity. Most editors of Public Health journals are affiliated with institutions in the Southeast and South regions as well as some states in the Northeast, whereas the participation of researchers from the North and Central-West regions remains limited. A greater balance in the composition of journal editors, encompassing different regions and social groups, would have positive repercussions with regards to editorial practice. Such diversity would contribute to the recognition of works relevant to different contexts of existence and belonging and to the selection of reviewers with greater assessment capacity for the topics in question. However, we cannot overlook the complexity of editorial work: the initial screening of manuscripts is common practice in all fields and is necessary to the maintenance of thematic coherence and the adequate quality of texts; the peer review crisis is global and long-standing, aggravated by the difficulty in finding qualified, committed reviewers; bad and even unethical opinions exist and it is up to the editorial board to sift them out.

The fourth axis focuses on other epistemologies and knowledge. Openness to non-hegemonic and emerging methodologies is faced with barriers. Valuing new theoretical and methodological approaches without sacrificing quality and scientific integrity implies not only accepting diversity but also recognizing it as a producer of legitimate knowledge, essential to the very advancement of science.

Lastly, the fifth axis involves ethical and political commitments and necessary transformations. To advance this discussion, it is necessary to problematize the internal and external pressures that perpetuate inequalities and restrict diversity, hindering more substantive changes ^4^, and that legitimize the role of the international publishing market, its economic logic and “bibliometric rules” that reproduce the scientific hierarchy between central and peripheral countries ^5^. This discussion regards traditional models of science assessment, the negative impacts of productivism, and the urgent need for national policies that promote open science without additional costs to authors or their institutions ^6^. Diamond open access, which is adopted by most Public Health journals in Brazil and other Latin American countries, should be valued, as it enables the elimination of financial barriers and democratizes scientific knowledge.

We recognize the complexity of editorial work in our country, which is largely carried out in a voluntary, serious, committed manner. We should not disregard the advances and dedication of those who work in the mediation of science on a daily basis. The prior rejection of articles, for instance, is not a selective practice directed against certain fields or institutions, but a recurring procedure in any scientific journal. Caution is also needed when discussing the classification and rejection of studies as “local”: it is not about barring studies that have unique territories and cultural realities as their field or object, but rather texts that do not enter into dialogue with broader theories or contexts and thus run the risk of becoming self-referential and so limited that they do not go beyond the empirical perimeters of that which was studied.

What is essential, therefore, is the creation of open channels of dialogue and spaces for listening among authors, editors, reviewers, and scientific communities. Advancing equity and diversity in scientific publishing requires addressing historical inequalities, beginning with the diversity of the group of editors of the journals themselves. The challenge is to combine criticism and commitment, recognizing limits and responsibilities. Only then will it be possible to consolidate more plural, inclusive journals capable of reflecting the complexity of the societies that they seek to understand and transform.
