# Supplementary material for: Diversity in scientific publishing is a strategic contribution to plural science
Source: Cad Saude Publica. 2025 Dec 1;41(11):e00200825. doi: 10.1590/0102-311XEN200825 (PMC12688206; doi:10.1590/0102-311XEN200825)
Supplement: Translation

# Translation: Diversidade na publicação científica é contribuição estratégica para uma ciência plural

**DOI:** 10.1590/0102-311XPT200825

**Authors:** Luciana Dias de Lima, Suely Deslandes

**Affiliations:** 1 Escola Nacional de Saúde Pública Sergio Arouca, Fundação Oswaldo Cruz, Rio de Janeiro, Brasil.; 2 Instituto Nacional de Saúde da Mulher, da Criança e do Adolescente Fernandes Figueira, Fundação Oswaldo Cruz, Rio de Janeiro, Brasil.

“*Do que estamos falando?*” - foi com essa pergunta que CSP abriu a mesa *Diversidade e Inclusão na Publicação Científica* durante o seminário organizado pelo Fórum de Editores de Saúde Coletiva da Abrasco, realizado em São Paulo, Brasil, em 12 e 13 de junho de 2025.

A pergunta, aparentemente simples, nos conduziu a um debate urgente e complexo sobre a persistência de desigualdades estruturais que atravessam a produção e a comunicação científica. O tema não é periférico. Trata-se de pensar quem tem voz na ciência, quem decide o que é publicado e quais saberes são legitimados nos espaços editoriais, em sociedades diversas e desiguais.

A importância dessa discussão reside em compreender que a publicação científica não é apenas uma instância técnica de avaliação e difusão do conhecimento, tampouco um espaço neutro. Ela é também um campo de disputas simbólicas, sociais e políticas, no qual se naturalizam desigualdades de gênero, raça, classe, território e idioma ^1^. Reconhecer essas assimetrias é um passo essencial para transformá-las, no intuito de possibilitar práticas editoriais mais plurais e inclusivas. 

Cinco grandes eixos estruturam esse debate e trazem desafios para as revistas científicas. O primeiro diz respeito às dimensões estruturais da desigualdade, manifestas na exclusão sistemática de determinados grupos sociais e na barreira linguística imposta pela hegemonia do inglês. Isso limita a circulação da produção do Sul Global e subvaloriza conhecimentos em outros idiomas ^2^. 

O segundo eixo refere-se às desigualdades de gênero, raça e território, expressas na invisibilidade de pesquisadoras(es) negras(os), indígenas e trans ^3^ e na concentração geográfica da produção científica. No Brasil, tal concentração reflete a distribuição dos principais centros de pesquisa e programas de pós-graduação.

O terceiro eixo aborda o papel de editores e revisores, envolvidos nos processos de avaliação e decisão sobre o que é publicável. É preciso reconhecer que os comitês editoriais ainda apresentam baixa diversidade. A maior parte dos editores das revistas de Saúde Coletiva está vinculada a instituições das regiões Sudeste, Sul e de alguns estados do Nordeste, ao passo que a participação de pesquisadores do Norte e do Centro-oeste permanece restrita. Um maior equilíbrio na composição dos editores das revistas, contemplando diferentes territórios e grupos sociais, repercutiria positivamente no ambiente da prática editorial. Essa diversidade contribuiria tanto para o reconhecimento de trabalhos relevantes aos distintos contextos de existência e pertencimento, quanto para a seleção de avaliadores com maior capacidade de apreciação das temáticas em questão. Ao mesmo tempo, não podemos desconsiderar a complexidade do trabalho editorial: a triagem inicial de manuscritos é prática comum em todas as áreas, necessária para manter a coerência temática e a qualidade mínima dos textos; a crise do *peer review* é global e antiga, agravada pela dificuldade de encontrar avaliadores qualificados e comprometidos; pareceres ruins e até antiéticos existem, e cabe ao corpo editorial filtrá-los.

O quarto eixo se volta às outras epistemologias e conhecimentos. A abertura a metodologias não hegemônicas e emergentes enfrenta barreiras, e valorizar novas abordagens teórico-metodológicas sem renunciar à qualidade e à integridade científica implica não apenas aceitar a diversidade, mas também reconhecê-la como produtora de conhecimento legítimo e imprescindível ao próprio avanço da ciência. 

Por fim, o quinto eixo envolve os compromissos ético-políticos e as transformações necessárias nesse âmbito. Para avançar nesse debate, é necessário problematizar as pressões internas e externas que perpetuam desigualdades e restringem a diversidade, dificultando mudanças mais substantivas ^4^. Nesse sentido, cabe reforçar junto à comunidade científica o papel do mercado editorial internacional, suas lógicas econômicas e “regras bibliométricas”, que reproduzem a hierarquia científica entre países centrais e periféricos ^5^. Essa discussão remete aos modelos tradicionais de avaliação da ciência, aos impactos negativos do produtivismo e à premência de políticas nacionais que impulsionem a ciência aberta, sem custos adicionais aos autores ou às suas instituições ^6^. O acesso aberto diamante, adotado pela maioria dos periódicos da Saúde Coletiva do Brasil e demais países da América Latina, deve ser valorizado, por permitir a eliminação de barreiras financeiras e democratizar o conhecimento científico. 

Reconhecemos a complexidade do trabalho editorial em nosso país, que é realizado em grande parte de forma voluntária, séria e comprometida. Não devemos desvalorizar os avanços e a dedicação de quem atua cotidianamente na mediação da ciência. A recusa prévia de artigos, por exemplo, não é uma prática dirigida seletivamente a determinadas áreas ou instituições, mas um procedimento recorrente em qualquer revista científica. Também é preciso cuidado ao discutir a classificação e rejeição de estudos como “locais”: não se trata de barrar estudos que tenham territórios e realidades culturais únicas como campo ou objeto, mas os textos que não dialogam com teorias ou contextos mais amplos e assim correm o risco de se tornarem autorreferenciais e tão limitados que não ultrapassam os perímetros empíricos do que foi pesquisado.

O que se impõe, portanto, é a construção de canais abertos de diálogo e espaços de escuta entre autores, editores, pareceristas e comunidades científicas. Avançar na equidade e na diversidade da publicação científica exige enfrentar desigualdades históricas, a começar pela diversidade do grupo de editores das próprias revistas. O desafio é conjugar crítica e compromisso, reconhecendo limites e responsabilidades. Só assim será possível consolidar periódicos mais plurais, inclusivos e capazes de refletir a complexidade das sociedades que buscam compreender e transformar.

# Translation: La diversidad en las publicaciones científicas es una contribución estratégica para una ciencia plural

**DOI:** 10.1590/0102-311XES200825

**Authors:** Luciana Dias de Lima, Suely Deslandes

**Affiliations:** 1 Escola Nacional de Saúde Pública Sergio Arouca, Fundação Oswaldo Cruz, Rio de Janeiro, Brasil.; 2 Instituto Nacional de Saúde da Mulher, da Criança e do Adolescente Fernandes Figueira, Fundação Oswaldo Cruz, Rio de Janeiro, Brasil.

“*¿De qué estamos hablando?*” - con esta pregunta CSP inauguró la mesa redonda *Diversidad e Inclusión en la Publicación Científica*, durante el seminario organizado por el Foro de Editores de Salud Colectiva de la Abrasco, que tuvo lugar en São Paulo, Brasil, los días 12 y 13 de junio de 2025.

La pregunta, aparentemente sencilla, nos llevó a un debate urgente y complejo: la persistencia de las desigualdades estructurales que atraviesan la producción y la comunicación científica. El tema no es secundario. Se trata de pensar quién tiene voz en la ciencia, quién decide qué se publica y qué conocimientos se legitiman en los espacios editoriales en sociedades diversas y desiguales.

La importancia de este debate radica en comprender que la publicación científica no es solo una instancia técnica de evaluación y difusión del conocimiento. Tampoco es un espacio neutral. También es un campo de disputas simbólicas, sociales y políticas, donde se naturalizan las desigualdades de género, raza, clase, territorio e idioma ^1^. Reconocer estas asimetrías es un paso esencial para transformarlas con el fin de posibilitar prácticas editoriales más plurales e inclusivas.

Cinco grandes ejes estructuran este debate y plantean retos para las revistas científicas. El primero se refiere a las dimensiones estructurales de la desigualdad, que se manifiestan en la exclusión sistemática de determinados grupos sociales y en la barrera lingüística impuesta por la hegemonía del inglés. Esto limita la circulación de la producción del Sur Global y subestima los conocimientos en otros idiomas ^2^. 

El segundo eje se refiere a las desigualdades de género, raza y territorio, expresadas en la invisibilidad de los(as) investigadores(as) negros(as), indígenas y trans ^3^, y en la concentración geográfica de la producción científica. En Brasil, dicha concentración refleja la distribución de los principales centros de investigación y programas de posgrado.

El tercer eje aborda el papel de los editores y revisores involucrados en los procesos de evaluación y decisión sobre lo que se publica. Es necesario reconocer que los comités editoriales aún presentan una baja diversidad. La mayor parte de los editores de las revistas de Salud Colectiva están vinculados a instituciones de las regiones Sudeste, Sur y algunos estados del Nordeste, mientras que la participación de investigadores del Norte y del Centro-Oeste sigue siendo restringida. Un mayor equilibrio en la composición de los editores de las revistas, que contemple diferentes territorios y grupos sociales, repercutiría positivamente en el entorno de la práctica editorial. Esta diversidad contribuiría tanto al reconocimiento de trabajos relevantes para los diversos contextos de existencia y pertenencia, como a la selección de evaluadores con mayor capacidad para apreciar los temas considerados. Al mismo tiempo, no podemos ignorar la complejidad del trabajo editorial: la selección inicial de manuscritos es una práctica habitual en todas las áreas, necesaria para mantener la coherencia temática y la calidad mínima de los textos; la crisis de la revisión por pares es global y antigua, agravada por la dificultad de encontrar revisores cualificados y comprometidos; existen opiniones negativas e incluso poco éticas, y corresponde al cuerpo editorial identificarlas.

El cuarto eje se centra en otras epistemologías y conocimientos. La apertura a metodologías no hegemónicas y emergentes se enfrenta a obstáculos. Valorar nuevos enfoques teórico-metodológicos sin renunciar a la calidad y la integridad científica implica no solo aceptar la diversidad, sino también reconocerla como generadora de conocimiento legítimo e imprescindible para el propio avance de la ciencia. 

Por último, el quinto eje abarca los compromisos ético-políticos y las transformaciones necesarias. Para avanzar en este debate, es necesario cuestionar las presiones internas y externas que perpetúan las desigualdades y restringen la diversidad, dificultando cambios más significativos ^4^. En este sentido, se debe reforzar ante la comunidad científica el papel del mercado editorial internacional, su lógica económica y sus “reglas bibliométricas”, que reproducen la jerarquía científica entre países centrales y periféricos ^5^. Este debate remite a los modelos tradicionales de evaluación de la ciencia, a los impactos negativos del productivismo y a la urgencia de políticas nacionales que impulsen la ciencia abierta, sin costes adicionales para los autores o sus instituciones ^6^. El acceso abierto diamante, adoptado por la mayoría de las revistas de Salud Colectiva de Brasil y otros países de América Latina, debe valorarse, ya que permite eliminar las barreras financieras y democratizar el conocimiento científico. 

Reconocemos la complejidad del trabajo editorial en nuestro país, que se realiza en gran parte de forma voluntaria, seria y comprometida. No debemos menospreciar los avances y la dedicación de quienes trabajan a diario en la mediación de la ciencia. El rechazo previo de artículos, por ejemplo, no es una práctica dirigida selectivamente a determinadas áreas o instituciones, sino un procedimiento recurrente en cualquier revista científica. También hay que tener cuidado al discutir la clasificación y el rechazo de estudios como “locales”: no se trata de bloquear estudios que tengan territorios y realidades culturales únicas como campo u objeto, sino los textos que no dialogan con teorías o contextos más amplios y, por lo tanto, corren el riesgo de volverse autorreferenciales y tan limitados que no superan los perímetros empíricos de lo que se investigó.

Por lo tanto, lo que se impone es la construcción de canales abiertos de diálogo y espacios de escucha entre autores, editores, revisores y comunidades científicas. Avanzar en la equidad y la diversidad de la publicación científica exige enfrentar las desigualdades históricas, comenzando por la diversidad del grupo de editores de las propias revistas. El reto consiste en combinar la crítica y el compromiso, reconociendo los límites y las responsabilidades. Sólo de este modo será posible consolidar revistas más plurales, inclusivas y capaces de reflejar la complejidad de las sociedades que buscan comprensión y transformación.
